# Neurodevelopmental Outcomes in Children Born to Mothers Infected with SARS-CoV-2 During Pregnancy: A Narrative Review

**DOI:** 10.3390/jcm14176202

**Published:** 2025-09-02

**Authors:** Daniela Păcurar, Alexandru Dinulescu, Ana Prejmereanu, Alexandru Cosmin Palcău, Irina Dijmărescu, Mirela-Luminița Pavelescu

**Affiliations:** 1 Faculty of Medicine, “Carol Davila” University of Medicine and Pharmacy, 050474 Bucharest, Romania; daniela.pacurar@umfcd.ro (D.P.); ana.prejmereanu@rez.umfcd.ro (A.P.); alexandru-cosmin.palcau@drd.umfcd.ro (A.C.P.); irina.dijmarescu@umfcd.ro (I.D.); mirela.pavelescu@umfcd.ro (M.-L.P.); 2 Department of Pediatrics, Emergency Hospital for Children “Grigore Alexandrescu”, 011743 Bucharest, Romania; 3 Department of General Surgery, University Emergency Hospital of Bucharest, 050098 Bucharest, Romania

**Keywords:** SARS-CoV-2, COVID-19, pregnancy, neurodevelopment, infants, maternal infection, developmental delay, prenatal exposure

## Abstract

**Background**: The potential impact of maternal SARS-CoV-2 infection during pregnancy on the neurodevelopment of offspring has raised considerable concern. Emerging studies have evaluated various developmental domains in exposed infants, yet findings remain inconsistent. **Objective**: To synthesize current evidence regarding neurodevelopmental outcomes in infants born to mothers with confirmed SARS-CoV-2 infection during pregnancy. Methods: We conducted a narrative review following PRISMA guidelines. A literature search was performed in PubMed, Cochrane, and ScienceDirect using keywords including “COVID-19”, “pregnancy”, “neurodevelopment”, and “SARS-CoV-2”. Nineteen studies were included. Data were extracted regarding study design, sample size, timing of exposure, age at assessment, developmental tools used, and key findings. Study quality was assessed using the Newcastle–Ottawa Scale. **Results**: Among 19 included studies, 12 reported at least some neurodevelopmental delays, particularly in motor and language domains. However, these delays were generally mild, domain-specific, and often not statistically significant. Seven studies, most of which were high-quality and low-risk, reported no significant differences between exposed and unexposed groups. Assessment tools and follow-up durations varied widely, limiting comparability. **Conclusions**: Current evidence does not support a consistent association between in utero SARS-CoV-2 exposure and an unfavorable neurodevelopmental outcome up to 24 months. However, heterogeneity in methods and short-term follow-up warrant further high-quality longitudinal research.

## 1. Introduction

The intrauterine environment plays a pivotal role in fetal development, including the processes of brain growth and maturation [1,2,3]. Maternal infections during pregnancy are well-documented contributors to negative neurodevelopmental outcomes in offspring, as pathogens or the maternal immune response can disrupt fetal brain development at critical stages [1,4,5,6,7,8]. Among the diverse etiologies that may impair fetal and early neurodevelopment—including metabolic, genetic, endocrine, and structural disorders, congenital infections represent a significant and potentially preventable cause of adverse outcomes [9,10,11,12,13,14]. Numerous viral infections, such as cytomegalovirus (CMV), Epstein–Barr virus (EBV), Zika virus (ZIKV), and influenza, have been associated with a spectrum of neurodevelopmental sequelae, including sensorineural hearing loss, intellectual disability, motor dysfunction, and autism spectrum disorders (ASD) [15,16,17,18,19,20,21]. CMV, for instance, is the most common congenital viral infection and a leading cause of neurodevelopmental disability, while prenatal exposure to maternal influenza has been linked to increased risks of schizophrenia and neurocognitive impairments in later life, although this statement needs more reliable data [20,22,23,24].

In this context, the emergence of SARS-CoV-2 as a novel coronavirus with global reach has raised concerns about its potential impact on children’s neurodevelopment [25,26,27]. Although vertical transmission of SARS-CoV-2 appears to be rare, the maternal immune activation (MIA) associated with COVID-19, characterized by systemic inflammation, cytokine release, and placental pathology, may represent a mechanism by which SARS-CoV-2 prenatal exposure could affect the developing fetal brain [26,28,29,30,31,32]. Furthermore, the pandemic has brought additional stressors such as altered prenatal care, maternal mental health issues, and social isolation, all of which may compound neurodevelopmental risks [33,34,35].

COVID-19 infection in pregnancy is a known factor for preterm birth, especially if the infection occurs in the third trimester or is a severe one [36,37,38]. There were also other complications described in COVID-19 pregnancy, such as an increased risk of hypertensive disorders of pregnancy, gestational diabetes, or an increased rate of neonatal intensive care unit (NICU) admission [39,40,41,42]. One important aspect is that the mRNA COVID-19 vaccine administered before pregnancy was not associated with an increased risk of any of those complications [43,44].

While initial concerns focused on obstetric complications such as preterm birth and vertical transmission, attention has increasingly turned to potential long-term consequences for offspring, particularly in the domain of neurodevelopment [43,44,45]. Recent reviews have begun to explore the early neurodevelopmental trajectories of children born to mothers infected with SARS-CoV-2 during pregnancy, with some reports suggesting subtle delays in motor, language, or social-emotional development during infancy [26,46]. It remains critical to disentangle the direct effects of the virus from indirect influences such as maternal emotional dysregulation and psychosocial stress during the pandemic, including social isolation and healthcare disruption, which may have further contributed to altered mother–infant interactions and long-term neurodevelopmental impact [47,48,49]. However, findings remain inconclusive, and methodological heterogeneity, regarding timing of maternal infection, severity of disease, gestational age at exposure, and follow-up duration, limits the generalizability of available evidence.

This review aims to synthesize current evidence on neurodevelopmental outcomes in children with in utero exposure to SARS-CoV-2. By exploring current epidemiologic findings, we seek to highlight both the knowns and unknowns in this evolving field and underscore the need for long-term, high-quality follow-up studies to fully understand the impact of COVID-19 on the next generation.

## 2. Materials and Methods

This study was conducted as a narrative review, with elements of systematic methodology applied to ensure rigor and transparency. A comprehensive literature search was performed in databases such as PubMed and Cochrane using predefined keywords. The selection process followed PRISMA principles, and study quality was assessed using the Newcastle–Ottawa Scale (NOS).

### 2.1. Inclusion Criteria

- Peer-reviewed observational studies (cohort or case–control).
- Neurodevelopment assessed via standardized tools (e.g., ASQ 3, Bayley scales) or clinical examination during infancy or early childhood (≤24 months), standardized parent-reported questionnaires (e.g., Ages and Stages Questionnaire), or direct neurological clinical examination during infancy or early childhood (≤24 months).

### 2.2. Exclusion Criteria

- Narrative reviews, animal studies (except mechanistic discussion), case reports without neurodevelopmental follow-up.
- Studies reporting exclusively biological outcomes (e.g., cytokine profiles, epigenetic changes) without separate reporting of standardized neurodevelopmental assessments.

### 2.3. Search Strategy, Selection and Data Extraction

**Figure 1:**
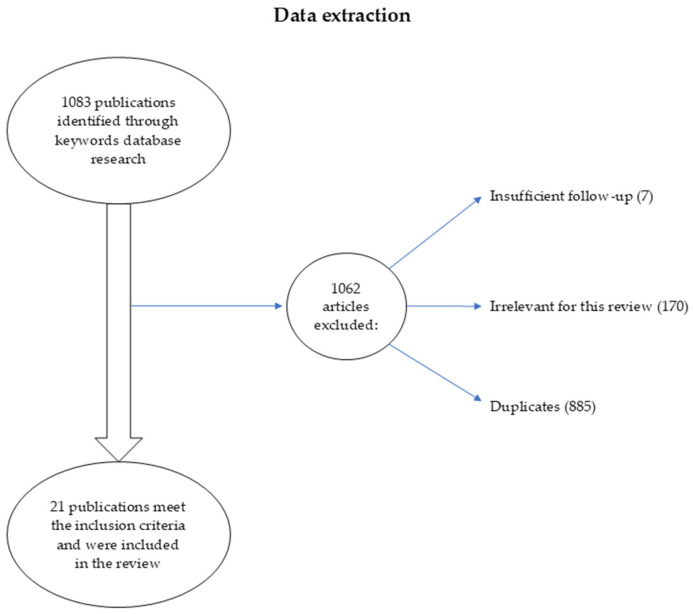
Data extraction.

A comprehensive literature search was conducted using PubMed, Cochrane Library, and ScienceDirect databases up to July 2025. The following keywords were used: (“SARS-CoV-2” OR “COVID-19”) AND (“pregnancy” OR “maternal infection”) AND (“neurodevelopment” OR “neurodevelopmental outcomes” OR “infant development”). Data extraction included sample size, neurodevelopmental tools used, follow-up age, and key outcomes. The search yielded 1083 articles. After removing duplicates and screening titles and abstracts, the full texts of potentially relevant studies were reviewed. Borderline-eligible studies were excluded if they did not report standardized neurodevelopmental outcomes separately, if they focused exclusively on biological outcomes without clinical correlation, or if they involved animal models. A total of 21 studies met the final inclusion criteria and were included in the review (Figure 1).

### 2.4. Data Synthesis

Due to substantial heterogeneity across the included studies in terms of neurodevelopmental assessment tools, follow-up duration, outcome domains, and reporting formats, a meta-analysis was not feasible. Instead, a narrative synthesis was performed. Results were organized thematically according to developmental domains (e.g., motor, language, cognitive, and social-emotional) and by the type and timing of maternal SARS-CoV-2 exposure. Consistencies and discrepancies across studies were qualitatively assessed, with attention to study quality, sample size, and methodological rigor as evaluated by the Newcastle–Ottawa Scale, interpreted as low risk of bias: 7–9; moderate risk of bias: 4–6 and high risk of bias: 0–3 (Table 1) [50].

Among the 21 included studies, 9 were rated as low risk of bias, 11 as moderate, and 1 as high risk. Studies with high total scores (e.g., Jaswa et al., Vrantsidis et al., Shuffrey et al.) were typically large, prospective cohorts with appropriate comparators and robust outcome assessment [51,66,67]. Studies rated as moderate risk tended to have smaller sample sizes, limited follow-up durations, or used less validated assessment tools.

## 3. Results

A total of 21 studies met the inclusion criteria for this narrative review, evaluating neurodevelopmental outcomes in infants born to mothers infected with SARS-CoV-2 during pregnancy. The sample sizes of the exposed groups ranged from 9 to 555, and neurodevelopmental assessments were performed at ages ranging from 6 weeks to 24 months postpartum. The most frequently used assessment tool was the Ages and Stages Questionnaire (ASQ-3). Other instruments used were the Denver Developmental Screening Test, General Movement Assessment (GMA), Motor Optimality Scores Revised (MOS-R), Neonatal Behavioral Assessment Scale (NBAS), Age and Stage Questionnaire Social-Emotional (ASQ:SE-2), Van Wiechen Scheme, International Statistical Classification of Diseases and Related Health Problems, Tenth Revision (ICD-10), Survey of Well-Being of Young Children (SWYC), Baby Pediatric Symptom Checklist (BPSC), and various clinical or parent-report measures.

Based on Table 2, 13 out of 21 studies (62%) reported at least some neurodevelopmental delays among SARS-CoV-2-exposed infants. These delays were most frequently observed in motor, language, and socioemotional domains, though the clinical significance and persistence of these delays varied considerably across studies.

### 3.1. Motor Development Findings

Motor development was the most commonly affected domain, with 11 studies reporting motor-related concerns. Shuffrey et al. (2021) found that both exposed and unexposed infants born during the pandemic had significantly lower scores in gross motor and fine motor domains compared to pre-pandemic cohorts, suggesting broader pandemic-related effects beyond direct viral exposure [51]. Several studies specifically identified fine motor delays: Cheng et al. (2021) reported significantly lower fine motor scores (*p* = 0.03) in exposed infants at 8–10 months, while Liu et al. (2022) found higher rates of fine motor abnormalities in exposed versus unexposed groups (15.2% vs. 2.1%; *p* = 0.02) [53,60]. Studies utilizing more sensitive motor assessment tools detected subtle neuromotor differences. Martinez et al. (2023) and Aldrete-Cortez et al. (2022), both using the Motor Optimality Score-Revised (MOS-R), found significantly lower median scores in exposed infants (23 vs. 25, *p* < 0.001; and 21 vs. 25, *p* = 0.002, respectively) [59,62]. Martinez et al. additionally reported that 16 exposed infants had MOS-R scores < 20 versus 0 controls (*p* < 0.001), and 13 exposed versus 0 controls showed developmental delays at 6–8 months clinical examination [62]. Kehdi et al. (2025) observed that 40% had motor delays at 6 months, this percentage increasing to 64.3% at 24 months, with specific cord blood cytokines correlating with motor delays (IL-6, IL-8, IL-17, and IL-1β) [70].

### 3.2. Communication and Language Development Findings

Communication delays were reported in several studies, with varying degrees of severity. Silva et al. (2025) found that 22 of 41 exposed children (53%) performed below the cutoff value in communication domains at 18 months [69]. Hill et al. (2024) reported lower ASQ-3 scores in communication domains among children exposed to severe maternal SARS-CoV-2 infection (*p* < 0.005), with findings correlating with specific cytokine profiles and epigenetic markers [68]. Berg et al. (2025), in one of the largest cohorts (*N* = 1446, 555 exposed), found no group differences in total ASQ scores, but noted increased risk of scores below cutoff among those exposed to severe maternal COVID-19 (16.0% vs. 6.1%; OR 3.57; 95% CI, 1.14–11.24) [71]. Finally, the only study rated as high risk of bias, Rood et al. (2023), suggested a mild delay but lacked a control group and included only 14 infants, limiting its reliability [65].

### 3.3. Socioemotional and Behavioral Outcomes

Several studies examined socioemotional development with mixed findings. Silva et al. (2023) reported increased odds of socioemotional developmental delay (OR = 4.0; *p* = 0.02) and inflexibility (OR = 14.0; *p* = 0.02) in exposed infants [63]. Ayesa-Arriola et al. (2023) found lower affectionate response scores in exposed groups (*p* = 0.009), particularly among those exposed in the third trimester (*p* = 0.043) [64]. Roffman et al. (2021) noted high irritability in exposed infants (*p* = 0.015) [52].

### 3.4. Timing of Maternal Infection

The trimester of maternal infection showed variable associations with outcomes. Ayed et al. (2022) found a higher risk of developmental delays with first-trimester (OR: 8.2, *p* = 0.039) and second-trimester (OR: 8.1, *p* = 0.001) infections compared to third-trimester exposure, but Ayesa-Arriola et al. (2023) reported more pronounced effects in third-trimester exposures, particularly for affectionate response measures (*p* = 0.043) [57,64]. It should be noted that more than a quarter (6/21) of the studies included had subjects only from the 3rd trimester infection, and three studies did not report this variable.

### 3.5. Disease Severity

Disease severity appeared to influence outcomes in some studies. Berg et al. (2025) specifically found increased risk only among infants exposed to severe maternal COVID-19 [71]. Hill et al. (2024) focused exclusively on severe maternal infections and reported significant developmental impacts with biological correlates [68].

### 3.6. Studies Reporting No Significant Differences

Seven studies reported no significant neurodevelopmental differences between exposed and unexposed groups. These included some of the largest and highest-quality studies: Jaswa et al. (2024) with 2003 participants (217 exposed) followed to 12–24 months, Vrantsidis et al. (2024) with 896 participants (96 exposed) followed to 6–24 months, and Wu et al. (2021) with 135 participants (57 exposed) assessed at 3 months [54,66,67]. These studies were notable for their larger sample sizes, appropriate control groups, and adjustment for multiple confounders, including maternal comorbidities, socioeconomic status, gestational age, and infant characteristics.

### 3.7. Adjustment for Confounders

Adjustment for confounders varied substantially across studies. Higher-quality studies typically adjusted for key variables, including gestational age, birthweight, maternal age, education, socioeconomic status, and infection characteristics. Studies with limited or no confounder adjustment showed more pronounced associations, raising questions about residual confounding.

## 4. Discussion

This narrative review synthesizes current evidence regarding neurodevelopment in infants born to mothers infected with SARS-CoV-2 during pregnancy, incorporating quality assessment using the Newcastle–Ottawa Scale to contextualize findings. While methodological heterogeneity presents interpretive challenges, emerging patterns regarding gestational timing, infection severity, and biological mechanisms warrant deeper clinical and scientific consideration alongside careful evaluation of study design limitations.

### 4.1. Overall Pattern of Findings and Study Quality Considerations

While 67% of studies reported some neurodevelopmental concerns, this proportion requires careful interpretation within the context of study quality and design. The highest-quality studies with low risk of bias (Jaswa et al., Vrantsidis et al., Shuffrey et al.) consistently reported no major developmental differences between exposed and unexposed groups [51,66,67]. This pattern suggests that apparent associations in lower-quality studies may reflect methodological limitations rather than true causal relationships.

The domains most frequently affected, motor development, communication, and socioemotional function, align with areas particularly vulnerable to early disruption and most readily assessed in infancy. However, the transient and mild nature of most reported delays, combined with their absence in well-controlled studies, suggests these findings may not represent clinically significant or persistent developmental alterations in the majority of exposed infants.

Overall, this review supports the statement that SARS-CoV-2 infection during pregnancy is not consistently associated with clinically significant neurodevelopmental delays in early infancy, but it remains essential to distinguish between the effects of being born during the pandemic and direct intrauterine exposure to SARS-CoV-2. Some delays may reflect broader societal disruptions rather than biological effects of the virus.

### 4.2. Gestational Timing of SARS-CoV2 Infection

The timing of maternal SARS-CoV-2 infection during pregnancy emerges as a critical determinant of neurodevelopmental risk, with evidence suggesting trimester-specific vulnerabilities that align with established principles of fetal brain development.

First-trimester exposure shows the strongest association with developmental delays, consistent with this period’s critical role in neural tube closure, neurulation, and establishment of basic brain architecture [72,73]. Ayed et al. (2022) provided compelling evidence for this vulnerability window, reporting dramatically increased odds ratios for developmental delays with first-trimester exposure (OR: 8.2, *p* = 0.039) [57]. During the first trimester, the blood–brain barrier is still developing, potentially allowing greater cytokine penetration into fetal neural tissue [74]. Established literature describes that first-trimester maternal immune activation affects fundamental neurodevelopmental processes, including neurogenesis, neuronal migration, and synaptic pruning [75,76]. The motor and reflex deficits specifically associated with first-trimester exposure may reflect disruption of brainstem and spinal cord development, which occurs primarily during weeks 4–8 of gestation. These structures are particularly vulnerable to inflammatory insults during their critical formation periods [77].

Second-trimester findings present complex findings, with Ayed et al. similarly reporting elevated risk (OR: 8.1, *p* = 0.001), while other studies suggest more subtle effects [57]. This period (weeks 13–26) encompasses peak cortical neurogenesis and the establishment of fundamental cortical architecture [78]. The apparent inconsistency in second-trimester findings across studies may reflect the diverse nature of developmental processes occurring during this extended period.

Third-trimester exposure presents the most intriguing findings, with studies reporting both protective and adverse effects. Ayesa-Arriola et al. (2023) found reduced affectionate response specifically in third-trimester exposed infants (*p* = 0.043), while several studies focusing exclusively on third-trimester exposure reported relatively preserved outcomes [64]. This apparent paradox may reflect the dual nature of late pregnancy immune activation. While the fetal brain is more mature and potentially more resistant to inflammatory disruption, this period encompasses critical synaptogenesis, circuit refinement, and the establishment of neurotransmitter systems crucial for social-emotional development [79].

### 4.3. Infection Severity

Studies focusing on mild to moderate maternal infections generally report reassuring outcomes. Berg et al. (2025), in their large cohort study (*N* = 1446), found no group differences in overall ASQ scores when analyzing the full spectrum of infection severity [71]. This suggests that the typical maternal inflammatory response to mild SARS-CoV-2 infection may be insufficient to significantly disrupt fetal neurodevelopment. The predominance of mild infections in many cohorts (often 80–95% of cases) likely contributes to the generally reassuring overall findings.

Neurodevelopmental effects are more consistently reported in studies examining severe maternal infections. Berg et al. specifically identified increased risk of developmental concerns only among infants exposed to severe maternal COVID-19 (16.0% vs. 6.1%; OR 3.57; 95% CI, 1.14–11.24) [71]. This finding suggests a dose–response relationship between maternal inflammatory burden and fetal neurodevelopmental risk. Hill et al. (2024) provided crucial mechanistic insights by focusing exclusively on severe maternal infections and demonstrating correlations between infant DNA methylation patterns and neurodevelopmental outcomes with specific cytokine profiles. Their finding of lower ASQ-3 scores in multiple domains (communication, problem solving, and personal-social; *p* < 0.005) coupled with epigenetic evidence provides the strongest support for biologically mediated effects [68].

### 4.4. Maternal Immune Activation

The concept of MIA provides a unifying framework for understanding SARS-CoV-2 effects on fetal neurodevelopment. Rather than requiring direct viral invasion of fetal neural tissue, MIA suggests that the maternal inflammatory response itself, can disrupt fetal brain development through shared inflammatory pathways [80]. This mechanism has been demonstrated across diverse maternal infections, from influenza to bacterial infections, suggesting common downstream effects despite different initial triggers [18,81]. The MIA model explains why SARS-CoV-2 effects on neurodevelopment show similarities to those observed with other prenatal infections, despite the virus’s unique characteristics. The key mediators are pro-inflammatory cytokines (such as IL-6, TNF-α, and IL-1β) that can cross the placenta and blood–brain barrier, triggering neuroinflammation in the developing fetal brain [31]. This neuroinflammation can then disrupt critical developmental processes, including neurogenesis, neuronal migration, synaptic formation, and myelination, regardless of whether the original pathogen directly infected neural tissue [76].

Kehdi et al. (2025) provided direct evidence of cytokine-mediated mechanisms, demonstrating that specific cord blood cytokines (IFN-γ, TNF-α, IL-6, IL-8, IL-17, IL-1β, CXCL10) correlated with domain-specific developmental delays [70]. The specificity of these correlations suggests that different cytokine profiles may preferentially affect distinct neurodevelopmental domains. Pro-inflammatory cytokines like TNF-α and IL-6 have established roles in disrupting neurogenesis and synaptic development, while chemokines like CXCL10 may affect microglial activation and neuroinflammation [82,83,84,85].

Beyond direct cytokine effects, SARS-CoV-2 infection can cause placental pathology, including thrombosis, infarction, and chronic villitis [86]. Liu et al. (2022) specifically linked fine motor abnormalities in exposed infants to placental hypoxia and ischemia, suggesting that vascular-mediated effects may be as important as direct inflammatory mechanisms [60].

Hill et al. (2024) provided groundbreaking evidence of epigenetic changes in infants exposed to severe maternal SARS-CoV-2 infection [68]. These DNA methylation alterations correlated with specific neurodevelopmental deficits, suggesting that maternal immune activation may program persistent changes in gene expression that influence long-term neurodevelopmental trajectories [87,88,89].

### 4.5. Specific Areas of Development Affected

Motor delays represent the most frequently reported and consistent finding across studies, appearing in 11 of 21 included studies. This consistency may reflect both the early emergence and relatively straightforward assessment of motor skills, but also suggests genuine vulnerability of motor systems to prenatal inflammatory insults. The progression from gross motor delays detected by standard screening tools to fine motor deficits identified by more sensitive instruments (MOS-R, GMA) indicates that motor effects may be subtle but persistent. Martinez et al. (2023) and Aldrete-Cortez et al. (2022) both demonstrated that specialized motor assessment tools could detect deficits missed by standard screening instruments, with significantly lower median MOS-R scores in exposed groups (23 vs. 25, *p* < 0.001; and 21 vs. 25, *p* = 0.002, respectively) [59,62]. The basal ganglia and cerebellar systems, vulnerable to prenatal inflammatory insults, are integral to both motor control and higher-order cognitive functions, potentially predicting later learning difficulties and attention problems [90,91].

Communication delays, while less consistently reported across all studies, may represent more clinically significant long-term effects when present. Silva et al. (2025) found that 53% of exposed children performed below cutoff values in communication domains at 18 months, a proportion that substantially exceeds the typical population prevalence of language delays (10–15%) [69]. The correlation between communication delays and specific cytokine profiles (Kehdi et al., 2025) suggests that neuroinflammation may particularly affect perisylvian cortical regions critical for language development [70,92].

Social-emotional findings are reported in fewer studies. Ayesa-Arriola et al. (2023) documented reduced affectionate response, while Silva et al. (2023) reported increased inflexibility (OR = 14.0; *p* = 0.02) and socioemotional developmental delays (OR = 4.0; *p* = 0.02) [63,64]. These findings align with established research linking prenatal immune activation to autism spectrum disorders, social anxiety, and other social-emotional difficulties [93,94,95].

### 4.6. Methodological Heterogeneity Impact on Interpretation

#### 4.6.1. Assessment Tools and Their Clinical Implications

The choice of neurodevelopmental assessment tool significantly influenced findings and their clinical interpretation. Parent-reported questionnaires such as the ASQ-3, while cost-effective and widely used, may be influenced by recall bias, parental stress, and pandemic-related anxiety [96]. These tools have known limitations in sensitivity for mild to moderate delays, and maternal education level significantly influences their accuracy [97]. The high prevalence of ASQ-3 use across studies (12 of 21) may contribute to apparent consistency of findings while actually reflecting shared methodological limitations rather than true developmental effects.

In contrast, studies using more sensitive, clinician-administered tools like the Motor Optimality Score-Revised (MOS-R) or General Movement Assessment (GMA) detected subtle neuromotor differences that may have greater clinical significance. However, these tools require specialized training and are resource-intensive, limiting their use to smaller cohorts and potentially introducing selection bias [98,99,100].

Direct neurological examination, while valuable for detecting overt abnormalities, may miss subtle cognitive or socioemotional delays that become apparent only with standardized developmental assessments [101]. This methodological diversity substantially contributes to the heterogeneity of findings and complicates direct comparison of results across studies.

#### 4.6.2. Follow-Up Duration and Developmental Windows

The variation in follow-up duration (6 weeks to 24 months) represents another critical limitation with important clinical implications. Most studies focused on early infancy, when many neurodevelopmental domains are still emerging and may not yet manifest subtle deficits. Domains such as executive function, attention regulation, and complex social behaviors typically become measurable only in later childhood, meaning that current evidence may not capture the full spectrum of potential effects.

Furthermore, the timing of assessment relative to critical developmental windows varies across studies, making it difficult to determine whether observed differences represent persistent delays, transient perturbations, or normal developmental variation. This limitation is particularly important given that some prenatal exposures may have effects that only become apparent during school age or adolescence.

#### 4.6.3. Distinguishing Viral Effects from Pandemic Context

A particularly important finding emerges from studies that attempted to distinguish direct viral effects from broader pandemic-related impacts. Shuffrey et al. (2021) [51] provided crucial insight by comparing three groups: pre-pandemic infants, pandemic-born unexposed infants, and pandemic-born exposed infants. Their finding that both pandemic-born groups showed lower developmental scores compared to pre-pandemic cohorts, regardless of maternal infection status, highlights the challenge of attributing developmental differences specifically to SARS-CoV-2 exposure [51]. This observation suggests that many reported developmental concerns may reflect broader pandemic-related disruptions, including altered prenatal care, maternal mental health impacts, reduced social stimulation, and disrupted healthcare access rather than direct viral effects [102,103]. The failure of many studies to account for these contextual factors represents a significant limitation in interpreting current literature.

#### 4.6.4. Confounding and Study Quality Considerations

The inconsistent approach to confounder adjustment across studies represents a major limitation affecting interpretation. Key confounders, including preterm birth, small for gestational age status, maternal mental health, socioeconomic factors, and healthcare access disruption, were variably addressed. Large registry-based studies and well-funded cohorts generally provided more comprehensive adjustment, while smaller studies often presented unadjusted comparisons.

Where adjustment was limited or absent, observed associations, particularly modest developmental score differences, should be interpreted cautiously, given the potential for residual confounding. The observation that studies with more comprehensive confounder adjustment were less likely to report significant associations supports this concern.

Among the 21 included studies, the 9 studies rated as low risk of bias using the Newcastle–Ottawa Scale were predominantly large, prospective cohorts with appropriate control groups and robust outcome assessments. Notably, most studies rated as low risk (6 of 9) reported no significant neurodevelopmental differences, while studies reporting delays were more frequently rated as moderate or high risk due to limitations in sample size, control group quality, follow-up duration, or assessment methodology. The only study rated as high risk of bias, Rood et al. (2023), suggested a mild delay but lacked a control group and included only 14 infants, limiting its reliability [65].

### 4.7. Similar Studies

Our finding is in concordance with other studies: a meta-analysis comparing infants born during compared to those born before the pandemic published by Hessami et al. (2022) found no overall rise in neurodevelopmental impairment, except for communication delay regardless of maternal infection and among infants with confirmed prenatal SARS-CoV-2, only fine motor performance showed a notable increase in risk (OR ≈ 3.5) [104]. A systematic review conducted by Veloso et al. (2024) focusing on the first postnatal year reported that while most SARS-CoV-2-exposed infants achieved age-appropriate development, mild deficits emerged, particularly in gross and fine motor domains and early on, and language/social domains, especially by parental report tools such as ASQ 3, NBAS, and BPSC [105]. Another meta-analysis published by Jackson et al. (2024), which included four studies up to 11 months postpartum, found no statistically significant increased risk of delay in communication, motor, problem-solving, or social domains among term infants [106].

### 4.8. Limitations

This review has several limitations. First, there is a substantial heterogeneity among included studies regarding neurodevelopmental assessment tools, timing of evaluations, and definitions of delay. Second, most studies had short-term follow-up, typically ending before 24 months, precluding evaluation of later-emerging deficits in domains such as executive function, attention, and social behavior. Third, while some studies used standardized clinical assessments, many relied on parent-report tools, which may be subject to re-reporting bias.

Additionally, confounding factors such as maternal stress, preterm birth, or pandemic-related changes in healthcare access were variably controlled. Only one study distinguished between direct viral effects and indirect environmental consequences of the pandemic, by comparing 3 groups: pre-pandemic group and 2 pandemic groups exposed and non-exposed [51].

### 4.9. Future Directions

Future research should focus on large-scale, longitudinal cohort studies that follow children with in utero SARS-CoV-2 exposure into later childhood and adolescence, allowing for the assessment of cognitive, behavioral, and emotional outcomes beyond the early developmental window. Standardized neurodevelopmental assessment tools, consistent follow-up intervals, and appropriate control groups (including both pandemic-born but unexposed children and pre-pandemic cohorts) are essential to improving comparability across studies. Investigations should also explore potential modifiers such as the timing of maternal infection during pregnancy, maternal mental health, preterm birth, and infant sex.

## 5. Conclusions

Based on the Newcastle–Ottawa Scale analysis, current evidence does not indicate consistent neurodevelopmental harm from prenatal SARS-CoV-2 exposure. Most well-designed studies support normal developmental trajectories up to 24 months; however, emerging evidence suggests that specific subgroups, particularly those exposed to severe maternal illness or during vulnerable gestational periods, may be at increased risk for subtle developmental effects. Nonetheless, further high-quality longitudinal research is needed to track potential late-emerging outcomes, especially in cognitive, emotional, and behavioral domains during school age. While reassuring trends emerge from high-quality studies, the nuanced nature of prenatal exposures, direct viral effects versus indirect psychosocial or immunological impacts, underscores the complexity of this topic. Thus, continued surveillance and rigorous, harmonized research protocols remain essential.

## Figures and Tables

**Table 1:** Risk of Bias Assessment (Newcastle–Ottawa Scale).

Number	Study	Selection (Max 4)	Comparability (Max 2)	Outcome (Max 3)	Total Score	Risk Level
1	Shuffrey et al., 2021 [51]	4	2	3	9	Low
2	Roffman et al., 2021 [52]	3	1	2	6	Moderate
3	Cheng et al., 2021 [53]	3	1	1	5	Moderate
4	Wu et al., 2021 [54]	3	1	2	6	Moderate
5	Schuh et al., 2021 [55]	2	1	1	4	Moderate
6	Edlow et al., 2022 [56]	4	2	2	8	Low
7	Ayed et al., 2022 [57]	3	2	2	7	Low
8	Buonsenso et al., 2022 [58]	3	1	1	5	Moderate
9	Aldrete-Cortez et al., 2022 [59]	3	1	2	6	Moderate
10	Liu et al., 2022 [60]	3	1	2	6	Moderate
11	Martenot et al., 2022 [61]	3	1	1	5	Moderate
12	Martinez et al., 2023 [62]	4	1	2	7	Low
13	Silva et al., 2023 [63]	3	1	2	6	Moderate
14	Ayesa-Arriola et al., 2023 [64]	2	1	2	5	Moderate
15	Rood et al., 2023 [65]	2	0	1	3	High
16	Vrantsidis et al., 2024 [66]	4	2	3	9	Low
17	Jaswa et al., 2024 [67]	4	2	3	9	Low
18	Hill et al., 2024 [68]	4	2	2	8	Low
19	Silva et al., 2025 [69]	3	1	2	6	Moderate
20	Kehdi et al., 2025 [70]	4	2	3	9	Low
21	Berg et al., 2025 [71]	4	2	3	9	Low

Note: Risk levels were defined as Low (7–9), Moderate (4–6), or High (0–3).

**Table 2:** Consolidated characteristics of included studies.

Study	Design/N (Exposed)	Maternal Infection Timing (Trimester)	Maternal Disease Severity	Neurodevelopment Tool	Follow-Up Age	Key Findings	Adjustment for Confounders
Shuffrey et al., 2021 [51]	Cohort/*N* = 317 (114 exposed)	1st: *n* = 42, 2nd: *n* = 90, 3rd: *n* = 67;)	34% asymptomatic, 62% mild/moderate, 4% severe	ASQ-3	6 months	No major neurodevelopmental differences in infants born during the pandemic (exposed vs. non-exposed), but those born during the pandemic had significantly lower scores on gross motor, fine motor, and personal-social subdomains when compared to a cohort born prior to the onset of the pandemic.	Yes—adjusted for maternal education, race/ethnicity, infant sex, GA, mode of delivery, NICU admission
Roffman et al., 2021 [52]	Cohort/*N* = 34 (19 exposed)	Not reported (NR)	NR	ASQ-3, BPSC	12 months	Gross motor delay and high irritability (*p* = 0.015).	NR
Cheng et al., 2021 [53]	Cohort/*N* = 18 (9 exposed)	All in the 3rd trimester	100% mild/moderate	ASQ-3	8–10 months	The infants from SARS-CoV-2-exposed mothers had lower scores in communication, gross movement, fine movement, problem solving, and personal-social domains, but only fine motor movement was significantly lower (*p* = 0.03).	NR
Wu et al., 2021 [54]	Cohort/*N* = 135 (57 exposed)	1st: *n* = 0, 2nd: *n* = 4, 3rd: *n* = 53	86% mild/moderate, 14% severe	ASQ-3, ASQ:SE-2	3 months	No significant neurodevelopmental differences between exposed and unexposed groups.	Yes—adjusted for mother–infant separation, low birth weight (LBW), infant sex, preterm birth, NICU admission, breastfeeding at 3 months
Schuh et al., 2021 [55]	Cohort/*N* = 15 (15 exposed)	NR	100% severe	ASQ-3	6 months	All of them were reported as normal development.	NR
Edlow et al., 2022 [56]	Cohort/*N* = 7772 (222 exposed)	1st: *n* = 1, 2nd: *n* = 61, 3rd: *n* = 160	NR	ICD-10 codes	12 months	Maternal SARS-CoV-2 positivity during pregnancy was associated with a greater rate of neurodevelopmental diagnoses (Odds ratio (OR) = 1.86, *p* = 0.04).	Yes—adjusted for maternal age, race/ethnicity, insurance status, gestational age (GA), birthweight, infant sex,
Ayed et al., 2022 [57]	Cohort/*N* = 298 (298 exposed)	1st: *n* = 5, 2nd: *n* = 20, 3rd: *n* = 273	39.5% asymptomatic, 53.6% mild/moderate, 6.9% severe	ASQ-3	10–12 months	The rate of development delays was 10%. The risk of developmental delays was higher with first-trimester (OR: 8.2, *p* = 0.039) and second-trimester maternal SARS-CoV-2 infections (OR: 8.1, *p* = 0.001) than with third-trimester.	Yes—adjusted for maternal age, maternal infection timing, GA, birthweight, infant sex, parental education and type of feeding in the first 6 months
Buonsenso et al., 2022 [58]	Cohort/*N* = 199 (199 exposed)	1st: *n* = 6, 2nd: *n* = 6, 3rd: *n* = 187	57.6% asymptomatic, 36.9% mild/moderate, 5.5% severe	Clinical exam	3–6-9–12 months	All of them were reported as normal neurological development.	NR
Aldrete-Cortez et al., 2022 [59]	Cohort/*N* = 56 (28 exposed)	All in 3rd trimester	100% mild/moderate	GMA	3–5 months	The exposed group had a significantly reduced total MOS-R; the median was lower in the exposed group (21 vs. 25, *p* = 0.002).	Yes—adjusted for maternal/infant characteristics differing between groups: maternal age, marital status, education level, preeclampsia, hypothyroidism, gestational diabetes, GA, infant sex, type of delivery, Hyperbilirubinemia, APGAR score, days of hospitalization, birthweight, length at birth, birth head circumference
Liu et al., 2022 [60]	Cohort/*N* = 98 (31 exposed)	All in the 3rd trimester	100% mild/moderate	Denver II	9 months	Fine motor abnormalities higher in the exposed group (15.2% vs. 2.1%; *p* = 0.02).	Yes—adjusted using for age, sex, infection status
Martenot et al., 2022 [61]	Cohort/*N* = 24 (24 exposed)	All in the 3rd trimester	4% asymptomatic, 96% symptomatic (severity NR)	ASQ-2	10 months	All of them were reported as normal neurological development.	NR
Martinez et al., 2023 [62]	Cohort/*N* = 239 (124 exposed)	1st: *n* = 17, 2nd: *n* = 37, 3rd: *n* = 70	14.5% asymptomatic, 67.7% mild/moderate, 17.7% severe	GMA, MOS-R, clinical exam	3–8 months	Suboptimal neuromotor development in exposed infants. The median of MOS-R was lower in the exposed group (23 vs. 25, *p* < 0.001), and 16 exposed infants had MOS-R scores <20 vs. 0 controls (*p* < 0.001). At 6–8 months clinical exam, 13 exposed vs. 0 controls had developmental delay.	Yes—adjusted for COVID-19 severity, trimester of infection, neonatal and maternal comorbidities, fetal sex, maternal age, maternal fever during COVID-19 and preterm birth
Silva et al., 2023 [63]	Cross-sectional study, *N* = 54 (27 exposed);	NR	NR	SWYC	From 1 to 12 months	Motor developmental delay in COVID-19-exposed infants (OR = 6.3, *p* = 0.01); socioemotional developmental delay (OR = 4.0; *p* = 0.02); Inflexibility (OR = 14.0; *p* = 0.02); Parental concerns about behavior, learning, or development of the infant (OR = 9.7; *p* = 0.01).	Yes—adjusted for sex, GA and family context.
Ayesa-Arriola et al., 2023 [64]	Cohort/*N* = 42 (21 exposed)	1st: *n* = 3, 2nd: *n* = 8, 3rd: *n* = 10	95.2% mild/moderate, 4.8% severe	NBAS (0–3 mo)	6 weeks	Lower affectionate response in the exposed group (*p* = 0.009), especially those exposed in the third trimester (*p* = 0.043).	Yes—for maternal age, GA, trimester of infection, infant age at assessment and infant sex
Rood et al., 2023 [65]	Cohort/*N* = 13 (13 exposed)	All in 3rd trimester	46% mild/moderate, 54% severe	Van Wiechen Scheme	3 months	Follow-up similar to children born to COVID-19-negative mothers, mild neurodevelopmental delay in 2 (15.3%).	NR
Vrantsidis et al., 2024 [66]	Cohort/*N* = 896 (96 exposed)	1st: *n* = 21, 2nd: *n* = 45, 3rd: *n* = 30	1% asymptomatic, 74% mild/moderate, 25% severe	ASQ-3	6–24 months	No significant neurodevelopmental differences between the exposed and unexposed groups	Yes—for maternal comorbidities and household socioeconomic status
Jaswa et al., 2024 [67]	Cohort/*N* = 2003 (217 exposed)	1st: *n* = 122, 2nd: *n* = 42, 3rd: *n* = 53	NR	ASQ-3	12–24 months	No significant neurodevelopmental differences between exposed and unexposed groups, or by the trimester of infection in the exposed group.	Yes—for maternal age, race, education level, income, maternal generalized anxiety and depression symptoms at baseline.
Hill et al., 2024 [68]	Cohort/*N*= 30 (16 expoed)	1st: *n* = 0, 2nd: *n* = 4, 3rd: *n* = 12	100% severe	ASQ-3	12 months	Lower ASQ-3 scores in children exposed to severe SARS-CoV-2 maternal infection in communication, problem solving and personal-social domains (*p* < 0.005); correlations with cytokine profiles and epigenetic markers.	NR
Silva et al., 2025 [69]	Cohort/*N* = 41 (41 exposed)	1st: *n* = 5, 2nd: *n* = 9, 3rd: *n* = 27	NR	ASQ-3	18 months	22 (53%) children performed below the cutoff value in communication and 19 (46%) in gross motor coordination.	Yes—for maternal age, GA, type of delivery, sex, Apgar score, birth weight, the child’s need for prolonged hospitalization after birth, and trimester infection
Kehdi et al., 2025 [70]	Cohort/*N* = 41 (18 exposed)	All in 3rd trimester	55% mild/moderate, 55% severe	Bayley-III	6–24 months	At 6 and 24 months, up to 36% cognitive, 64% communication, and 57% motor delays were observed. Specific cord blood cytokines correlated with respective domain delays.	No adjustment for confounders; BSID-III scores corrected for gestational age
Berg et al., 2025 [71]	Cohort/*N* = 1446 (555 exposed)	1st: *n* = 63, 2nd: *n* = 278, 3rd: *n* = 214	95.5% mild/asymptomatic, 4.5% severe	ASQ-3	4 months	There was no group difference in ASQ total mean scores, but those exposed to severe maternal COVID-19 had an increased risk of total ASQ scores below the cutoff (exposed: 16.0% vs. unexposed: 6.1%; OR 3.57; 95% CI, 1.14–11.24).	Yes—for mother BMI, education, country of origin, maternal age, pre-pregnancy comorbidity, GA, APGAR, type of delivery, birthweight, type of feeding

## Data Availability

Data are contained within the article.

## Abbreviations

The following abbreviations are used in this manuscript:

COVID 19	Coronavirus disease 2019
SARS-CoV-2	Severe acute respiratory syndrome coronavirus 2
PRISMA	Preferred Reporting Items for Systematic reviews and Meta-Analyses
ASD	Autism spectrum disorders
CMV	Cytomegalovirus
EBV	Epstein–Barr virus
NOS	Newcastle–Ottawa Scale
OR	Odds ratio
ASQ-3	Ages and Stages Questionnaire
BPSC	Baby Pediatric Symptom Checklist
GMA	General Movement Assessment
MOS-R	Motor Optimality Scores Revised
NBAS	Neonatal Behavioral Assessment Scale
ICD-10	International Statistical Classification of Diseases and Related Health Problems, Tenth Revision
SWYC	Survey of Well-Being of Young Children
ASQ:SE-2	Age and Stage Questionnaire Social-Emotional
MIA	Maternal Immune Activation
LBW	Low birth weight
GA	Gestational age
OR	Odds ratio
